# Longitudinal Association Between Physical Exercise and Depressive Symptoms in Older Adults: The Prospective Explanatory Role of Loneliness and the Moderating Role of Cognitive Emotion Regulation

**DOI:** 10.3390/bs16071108

**Published:** 2026-07-03

**Authors:** Renjie Ma, Haozhen Li, Qiuhan Zhu

**Affiliations:** 1 School of Marxism, Zhengzhou University, Zhengzhou 450001, China; marenjie0501@gs.zzu.edu.cn; 2 School of Kinesiology and Physical Education, Zhengzhou University, Zhengzhou 450001, China; lihaozhen01@zzu.edu.cn

**Keywords:** physical exercise, depressive symptoms, loneliness, cognitive emotion regulation, two-wave prospective study, older adults

## Abstract

**Objective**: This study examined the prospective association between self-reported physical exercise and depressive symptoms among older adults, and further tested whether loneliness statistically accounted for this association and whether baseline cognitive emotion regulation strategies moderated the exercise–loneliness pathway. **Methods**: A two-wave prospective survey with a six-month interval was conducted among 980 community-dwelling older adults in Zhengzhou, China. Baseline data were collected in September 2024, and follow-up data were collected in March 2025. Physical exercise was assessed using the Physical Activity Rating Scale-3, depressive symptoms using the 10-item Center for Epidemiologic Studies Depression Scale, loneliness using the 8-item UCLA Loneliness Scale, and cognitive emotion regulation strategies using the short version of the Cognitive Emotion Regulation Questionnaire. Cross-lagged models were used to examine the residual prospective association between physical exercise and depressive symptoms. A two-wave prospective explanatory model was then tested to examine the role of follow-up loneliness, and moderated explanatory (half-longitudinal) analyses were conducted using baseline adaptive and maladaptive cognitive emotion regulation strategies as moderators. **Results**: Baseline physical exercise was significantly associated with lower depressive symptoms at follow-up after controlling for baseline depressive symptoms and covariates (β = −0.182, *p* < 0.001). In contrast, baseline depressive symptoms were not significantly associated with follow-up physical exercise (β = −0.016, *p* = 0.633). Baseline physical exercise was negatively associated with follow-up loneliness (β = −0.267, *p* < 0.001), and follow-up loneliness was positively associated with follow-up depressive symptoms (β = 0.324, *p* < 0.001). The indirect association through follow-up loneliness was significant (indirect effect = −0.087, 95% CI [−0.112, −0.065]). Baseline adaptive cognitive emotion regulation strategies strengthened the association between physical exercise and lower loneliness (interaction β = −0.076, *p* < 0.001), whereas baseline maladaptive strategies weakened this association (interaction β = 0.059, *p* = 0.004). **Conclusions**: Self-reported physical exercise was prospectively associated with fewer depressive symptoms among older adults six months later. This association was statistically accounted for, in part, by lower follow-up loneliness, and baseline cognitive emotion regulation strategies were associated with the strength of the exercise–loneliness association. Because this study used a two-wave observational design with the explanatory variable and outcome measured at the same follow-up wave, the findings should be interpreted as prospective associations rather than evidence of causal or temporal mediation.

## 1. Introduction

Depressive symptoms among older adults have become an increasingly important public health issue in the context of rapid population aging (Ganafa et al., 2025; Hong et al., 2025). In later life, depressive symptoms are associated not only with emotional distress, but also with reduced social functioning, poorer physical health, lower quality of life, and increased care needs (Ren et al., 2025). Because many older adults experience retirement, bereavement, declining physical capacity, and shrinking social networks, identifying modifiable factors that are associated with better psychological well-being is essential for developing feasible community-based prevention and intervention strategies.

Physical exercise is one such modifiable behavior. Compared with pharmacological or clinic-based interventions, physical exercise is relatively accessible, low-cost, and easy to implement in daily life. A growing body of research suggests that older adults who participate in physical exercise tend to report fewer depressive symptoms (Kaczorowska et al., 2024; Mammen & Faulkner, 2013; Pearce et al., 2022; Yang et al., 2025; Zhang et al., 2024). However, the interpretation of this association remains limited for two reasons. First, many previous studies have used cross-sectional designs, which cannot clarify whether physical exercise precedes lower depressive symptoms or whether older adults with fewer depressive symptoms are simply more likely to engage in exercise. Second, existing studies have often focused on whether physical exercise is associated with depressive symptoms while paying less attention to the psychosocial mechanisms through which this association may occur and the individual conditions under which it may be stronger or weaker (Kandola et al., 2019).

A longitudinal perspective is therefore needed to examine the temporal ordering between physical exercise and depressive symptoms. Physical exercise may serve as a behavioral context that supports regular routines, positive activity engagement, and social participation, all of which may be beneficial for mental health in later life. At the same time, depressive symptoms may reduce motivation, energy, and willingness to participate in physical activity. Thus, the association between exercise and depression may be potentially bidirectional (Choi et al., 2019). By using two-wave data, the present study first examined whether baseline physical exercise predicted depressive symptoms six months later after accounting for baseline depressive symptoms, and whether the reverse path from baseline depressive symptoms to later physical exercise was also evident.

Beyond residual prospective ordering, it is also important to understand why physical exercise may be associated with lower depressive symptoms. Loneliness is a theoretically meaningful explanatory factor. Loneliness refers to the subjective experience of insufficient or unsatisfactory social connection (Hawkley & Cacioppo, 2010). It is particularly relevant among older adults because later life is often accompanied by reductions in social roles, loss of close relationships, and changes in family structure. Persistent loneliness has been closely linked to depressive symptoms, as perceived social disconnection may increase negative affect, reduce perceived support, and reinforce withdrawal from social engagement (Damor et al., 2024; Liu et al., 2025; Santini et al., 2015). When exercise occurs in group or community settings, it may provide repeated opportunities for social contact, shared routines, informal communication, and a sense of group belonging (Sarikahya et al., 2024). However, because the present exercise measure did not assess the social setting of activity, this social-context interpretation should be regarded as a plausible explanation rather than a directly tested mechanism.

However, the mediating role of loneliness should be specified carefully. A strict longitudinal mediation model requires at least three measurement waves, with the predictor, mediator, and outcome assessed at separate time points (Cole & Maxwell, 2003). The present study used a two-wave design in which physical exercise was measured at baseline, while loneliness and depressive symptoms were measured at follow-up. Although baseline loneliness and baseline depressive symptoms were controlled, follow-up loneliness and follow-up depressive symptoms were assessed at the same wave. Therefore, this study does not claim to establish a definitive causal mediation process. Instead, it examines whether follow-up loneliness statistically accounts for the prospective association between baseline physical exercise and follow-up depressive symptoms. This approach is best understood as a two-wave prospective or half-longitudinal explanatory model.

In addition to examining loneliness as an explanatory factor, it is necessary to consider why the psychological correlates of physical exercise may differ across individuals. Exercise may create opportunities for social interaction in some settings, but such opportunities do not automatically translate into a reduced sense of loneliness. Whether older adults benefit emotionally from exercise-related experiences may depend on how they cognitively process them. Cognitive emotion regulation refers to the cognitive strategies individuals use to manage emotional responses to stressful or negative events (Aldao et al., 2010; Garnefski & Kraaij, 2006). Adaptive strategies, such as positive reappraisal, positive refocusing, putting into perspective, and refocus on planning, may help individuals interpret social experiences in a more constructive way (Larionow et al., 2025). In contrast, maladaptive strategies, such as rumination, self-blame, other-blame, and catastrophizing, may intensify negative interpretations and prolong distress.

Cognitive emotion regulation may be especially relevant to the association between physical exercise and loneliness. Physical exercise can provide an external opportunity for social connection when it occurs with others, but cognitive processing influences whether that opportunity is perceived as supportive, meaningful, or threatening. Older adults who frequently use adaptive cognitive emotion regulation strategies may be more likely to notice positive social cues, reinterpret minor interpersonal discomforts constructively, and maintain engagement with exercise groups. As a result, physical exercise may be more strongly associated with lower loneliness among these individuals. Conversely, older adults who rely more heavily on maladaptive strategies may dwell on ambiguous or negative social experiences, magnify minor interpersonal difficulties, or interpret neutral interactions as signs of rejection. For them, exercise-related social contact, when present, may be less likely to translate into a subjective sense of connection. Thus, baseline cognitive emotion regulation strategies may function as boundary conditions for the association between physical exercise and subsequent loneliness.

The present study used a two-wave prospective design to examine the association between self-reported physical exercise and depressive symptoms among community-dwelling older adults in Zhengzhou, China. This study aimed to address three research questions. First, does baseline physical exercise predict depressive symptoms six months later after controlling for baseline depressive symptoms and relevant covariates? Second, does follow-up loneliness partially explain the prospective association between baseline physical exercise and follow-up depressive symptoms after controlling for baseline loneliness and depressive symptoms? Third, do baseline adaptive and maladaptive cognitive emotion regulation strategies moderate the association between baseline physical exercise and follow-up loneliness?

Based on the above reasoning, the following hypotheses were proposed:

**H1.**  *Higher baseline physical exercise would be associated with lower depressive symptoms at follow-up after controlling for baseline depressive symptoms and relevant covariates.*

**H2.**  *Follow-up loneliness would partially account for the prospective association between baseline physical exercise and follow-up depressive symptoms after controlling for baseline loneliness and baseline depressive symptoms.*

**H3a.**  *Baseline adaptive cognitive emotion regulation strategies would strengthen the negative association between baseline physical exercise and follow-up loneliness.*

**H3b.**  *Baseline maladaptive cognitive emotion regulation strategies would weaken the negative association between baseline physical exercise and follow-up loneliness.*

**Figure 1:**
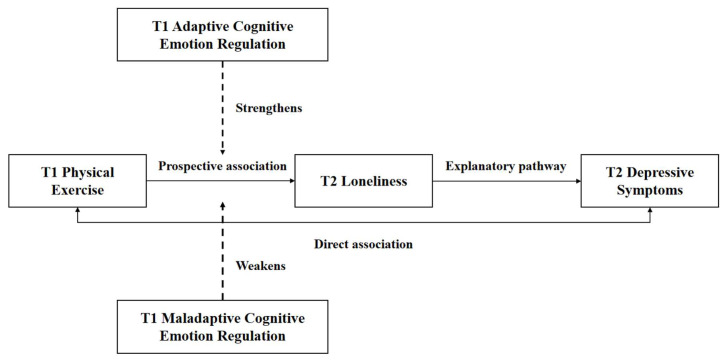
Concept of the two-wave prospective explanatory model.

The conceptual model is presented in Figure 1.

## 2. Participants and Methods

### 2.1. Participants

**Study Design.** This study used a two-wave prospective design to examine the association between self-reported physical exercise and depressive symptoms among community-dwelling older adults. Reporting follows the STROBE guidance for cohort studies, and the completed STROBE checklist is provided in the Supplementary Materials (Table S1). Because the physical-activity measure assessed overall exercise volume rather than the social setting of exercise, the exposure is referred to as physical exercise (rather than community or group-based exercise) throughout. The baseline survey was conducted in September 2024, and the follow-up survey was conducted six months later in March 2025. The six-month interval was selected to capture short- to medium-term changes in exercise participation, loneliness, cognitive emotion regulation, and depressive symptoms while reducing the likelihood that the follow-up interval would be too short to observe meaningful psychological variation. A six-month interval has also been adopted in intervention research on physical activity and psychosocial well-being among community-dwelling older adults, including outcomes such as depressive symptoms and loneliness (Hotterbeex et al., 2026).

**Setting.** Participants were recruited from urban communities in Zhengzhou, China. A multi-stage community-based sampling procedure was used. First, three urban districts in Zhengzhou were selected to represent different geographical and socioeconomic areas of the city. Second, one sub-district was selected from each district. Third, five community residents’ committees were selected from each sub-district. In total, participants were recruited from 15 urban communities, with an average of approximately 65 older adults per community. Finally, community workers and staff from community health service centers helped identify potentially eligible older residents and invited them to participate in the study. Because recruitment proceeded through community-based gatekeepers rather than a complete population register, the sample should be regarded as a community convenience sample, and the potential for sampling bias is acknowledged in the Limitations.

**Participants (Eligibility Criteria).** The inclusion criteria were as follows:

- aged 60 years or above;
- having lived in the selected community for at least six months;
- being able to understand and complete the questionnaire independently or with standardized assistance from trained research staff;
- providing written informed consent.

Participants were excluded if they had severe cognitive impairment, severe visual or hearing impairment that prevented questionnaire completion, or a previously diagnosed major psychiatric or neurological disorder that could substantially interfere with participation. The presence of a previously diagnosed major psychiatric or neurological disorder was identified through participant self-report and, where available, confirmation by community health records and community workers. Cognitive screening was conducted using the Mini-Mental State Examination (Folstein et al., 1975), applying education-specific cut-off scores to identify possible cognitive impairment (illiteracy ≤ 17, primary school ≤ 20, and junior high school or above ≤ 24) (Crum et al., 1993). Participants who did not meet the cognitive screening criteria were not included in the final survey.

**Participant Flow**. At baseline, 1350 older adults were invited to participate. Of these, 61 declined or were unreachable, yielding 1289 returned questionnaires. A further 45 were excluded for failing the cognitive screening and 44 for extensive missing data or logical inconsistencies, resulting in 1200 valid baseline responses. Over the six-month interval, 157 participants were lost to follow-up (85 relocated or lost contact, 42 declined the second wave, and 30 withdrew due to hospitalization or severe illness). During cross-wave data matching and quality control, an additional 63 responses were excluded (41 had unmatched identification codes, and 22 had more than 10% missing data on key study variables). Thus, the final analytic sample comprised 980 participants.

To examine potential attrition bias, baseline characteristics were compared between the 980 retained participants and those lost to follow-up using independent-samples t-tests and chi-square tests, with effect sizes reported. No significant differences were observed in age (t = 1.54, *p* = 0.124, Cohen’s d = 0.11), baseline physical exercise (t = 1.48, *p* = 0.139, d = 0.10), baseline depressive symptoms (t = 1.25, *p* = 0.212, d = 0.08), or baseline loneliness (t = 1.34, *p* = 0.181, d = 0.09). All effect sizes were trivial (d < 0.20), suggesting that attrition was unlikely to introduce serious systematic bias, although unmeasured attrition-related differences could not be completely excluded. Missing data within the analytic framework were handled using full-information maximum likelihood (FIML) estimation.

**Bias.** To reduce potential bias, standardized and validated instruments were administered under uniform procedures across both waves, trained research assistants followed a neutral protocol, unique identification codes ensured accurate matching of baseline and follow-up data, and attrition and common-method bias were examined (see Section 2.1 and Section 4.7).

All participants were informed of the purpose, procedures, voluntary nature, and confidentiality of the study before completing the questionnaire. Written informed consent was obtained from all participants. The study protocol was reviewed and approved by the Ethics Committee of Zhengzhou University and was conducted in accordance with the Declaration of Helsinki.

### 2.2. Sample Size Consideration (**Study Size**)

Because the present study tested prospective associations and explanatory pathways within a structural equation modeling (SEM) framework, sample size requirements were considered from the perspective of covariance structure modeling rather than single-equation regression. Following the procedure of MacCallum et al. (1996), an a priori power analysis for testing close fit (null RMSEA = 0.08 versus alternative RMSEA = 0.05) at α = 0.05 indicated that, given the degrees of freedom in the main models, a minimum of approximately 400 participants was required to achieve a power of 0.80.

Considering possible attrition over the six-month follow-up, the initial recruitment target was increased by approximately 20%. The final analytic sample of 980 participants substantially exceeded this minimum and provided excellent statistical power (>0.95) for evaluating the hypothesized pathways.

### 2.3. Measures (**Variables, Data Sources, and Measurement**)

#### 2.3.1. Demographic and Health-Related Variables

A structured questionnaire was used to collect demographic and health-related information. The demographic variables included gender, age, education level, living arrangement, and marital status. Health-related variables included smoking status, alcohol consumption, number of chronic diseases, and self-rated health status. In addition, participants reported their frequency of social activity and perceived interpersonal support. These variables were included as covariates because they may be associated with physical exercise, loneliness, and depressive symptoms in later life.

#### 2.3.2. Physical Exercise

Physical exercise was assessed using the Physical Activity Rating Scale-3 (Liang, 1994). This scale evaluates exercise participation based on three dimensions: exercise intensity, duration of each exercise session, and exercise frequency. Each dimension is scored using ordered response categories. The total exercise score is calculated using the following formula:

exercise score = intensity × (duration − 1) × frequency

In this scoring method, subtracting one from the duration score allows the lowest duration category to contribute a value of zero, reflecting minimal exercise participation. Higher scores indicate a greater overall level of physical exercise. The Chinese version of the PARS-3 has demonstrated adequate reliability and validity among older adult populations in prior studies (Liang, 1994). In the present study, physical exercise was assessed at both baseline and follow-up (Cronbach’s α = 0.82 and 0.84, respectively; observed range 0–75). Baseline physical exercise was used as the main predictor in the prospective models, while follow-up physical exercise was used in the cross-lagged analysis.

#### 2.3.3. Depressive Symptoms

Depressive symptoms were assessed using the 10-item Center for Epidemiologic Studies Depression Scale (Andresen et al., 1994; Chen & Mui, 2014). The CESD-10 measures the frequency of depressive symptoms experienced during the past week. Items are rated on a four-point scale ranging from 0 to 3. Two positively worded items are reverse-scored, and the total score is obtained by summing all items. Higher scores indicate more severe depressive symptoms. The CESD-10 includes one item assessing loneliness (“I felt lonely”); because this item conceptually overlaps with the loneliness measure, a sensitivity analysis re-estimating the models after removing this item was conducted (see Section 3.6).

In this study, depressive symptoms were measured at both baseline and follow-up. Baseline depressive symptoms were controlled in the prospective models to reduce confounding by prior depressive symptom levels. Follow-up depressive symptoms served as the main outcome variable. Internal consistency was good at both waves (Cronbach’s α = 0.86 and 0.87; observed range 0–26).

#### 2.3.4. Loneliness

Loneliness was measured using the 8-item UCLA Loneliness Scale (Hays & DiMatteo, 1987). The ULS-8 is a shortened version of the UCLA Loneliness Scale and assesses subjective feelings of social isolation and insufficient social connection (Bahadori et al., 2025). Participants rated each item on a four-point scale, with higher total scores indicating stronger loneliness.

Loneliness was measured at both baseline and follow-up. Baseline loneliness was included as a covariate in the explanatory models, and follow-up loneliness was examined as an explanatory variable linking baseline physical exercise to follow-up depressive symptoms. Because follow-up loneliness and follow-up depressive symptoms were measured at the same wave, the analysis was interpreted as a two-wave prospective or half-longitudinal explanatory model rather than as evidence of strict longitudinal mediation. Internal consistency was good at both waves (Cronbach’s α = 0.89 and 0.90; observed range 8–32).

#### 2.3.5. Cognitive Emotion Regulation Strategies

Cognitive emotion regulation strategies were assessed using the short version of the Cognitive Emotion Regulation Questionnaire (Garnefski & Kraaij, 2006). The CERQ-short measures cognitive strategies that individuals use to regulate emotional responses after experiencing negative or stressful events. It includes nine strategy dimensions: acceptance, positive refocusing, refocus on planning, positive reappraisal, putting into perspective, rumination, self-blame, other-blame, and catastrophizing.

Following previous research, these strategies were grouped into two broader categories. Adaptive cognitive emotion regulation strategies included acceptance, positive refocusing, refocus on planning, positive reappraisal, and putting into perspective. Maladaptive cognitive emotion regulation strategies included rumination, self-blame, other-blame, and catastrophizing. Mean scores were calculated separately for adaptive and maladaptive strategies, with higher scores indicating more frequent use of the corresponding type of strategy. Internal consistency was good for both composites at both waves (adaptive: Cronbach’s α = 0.88 and 0.89, observed range 10–50; maladaptive: α = 0.85 and 0.86, observed range 8–40). Baseline adaptive and maladaptive strategies were moderately negatively correlated (r = −0.386, *p* < 0.001), indicating that they captured related but distinct tendencies.

In the main conditional explanatory analysis, baseline adaptive and maladaptive cognitive emotion regulation strategies were used as moderators. This decision was made because cognitive emotion regulation strategies were conceptualized as relatively stable cognitive tendencies that may shape how older adults interpret and benefit from exercise-related social experiences. Using baseline moderators also helped reduce ambiguity caused by measuring the moderator and mediator at the same follow-up wave.

### 2.4. Data Collection Procedure

Data were collected by trained research assistants with backgrounds in public health, psychology, or physical education. Before data collection, all research assistants received standardized training on the study protocol, questionnaire administration, communication with older adults, informed consent procedures, and quality control requirements.

Both paper-and-pencil and online questionnaires were used; overall, 38% of participants completed paper-and-pencil questionnaires, and 62% completed online questionnaires. Paper questionnaires and interviewer assistance were prioritized for participants with limited familiarity with smartphone-based surveys, whereas online questionnaires were used when participants were able and willing to complete them independently. For participants with low literacy, visual difficulty, or difficulty writing, trained research assistants read the items aloud in a neutral manner and recorded the participants’ responses without providing suggestive explanations.

To match baseline and follow-up data while protecting personal privacy, participants were asked to generate a unique identification code based on personal information known only to them. The same coding rule was used at both waves. After each survey session, research assistants checked the questionnaires for completeness. Questionnaires with substantial missing data, patterned responses, or logical inconsistencies were flagged for further review. Data from paper questionnaires were entered by trained staff and cross-checked to reduce data entry errors.

### 2.5. Statistical Analysis (**Statistical Methods**)

All statistical analyses were conducted using SPSS 26.0 and Mplus 8.3. Statistical significance was evaluated using two-sided tests with an alpha level of 0.05.

First, descriptive statistics were calculated for demographic characteristics and key study variables. Means and standard deviations were reported for continuous variables, and frequencies and percentages were reported for categorical variables. Distributional properties were examined; the PARS-3 score was slightly right-skewed (skewness = 0.68, kurtosis = −0.15), consistent with its multiplicative scoring. To accommodate this minor non-normality, robust maximum likelihood estimation (MLR) with robust standard errors was used in all Mplus path models. Pearson correlation analyses were conducted to examine bivariate associations among physical exercise, loneliness, depressive symptoms, and cognitive emotion regulation strategies across the two waves; as a robustness check against the assumption of continuous scaling, Spearman rank-order correlations were also computed and yielded substantively identical patterns (e.g., baseline exercise with follow-up depressive symptoms, r_s = −0.44).

Second, a cross-lagged panel model was estimated to examine the residual prospective association between physical exercise and depressive symptoms. The model included autoregressive paths from baseline physical exercise to follow-up physical exercise and from baseline depressive symptoms to follow-up depressive symptoms. It also included cross-lagged paths from baseline physical exercise to follow-up depressive symptoms and from baseline depressive symptoms to follow-up physical exercise. This analysis was used to examine whether physical exercise prospectively predicted depressive symptoms after accounting for baseline depressive symptoms, and whether the reverse prospective association was also significant. Because the data were observational and limited to two waves, the cross-lagged model was interpreted as evidence of a residual prospective association rather than of temporal precedence, directional influence, or causal effect. A traditional two-wave cross-lagged panel model cannot separate stable between-person differences from within-person change.

Third, a two-wave prospective explanatory model was tested. Baseline physical exercise was specified as the predictor, follow-up depressive symptoms as the outcome, and follow-up loneliness as the explanatory variable. Baseline loneliness and baseline depressive symptoms were included as covariates. Demographic and health-related covariates were also controlled, including gender, age, education level, living arrangement, marital status, smoking status, alcohol consumption, number of chronic diseases, self-rated health status, social activity frequency, and interpersonal support. Bias-corrected bootstrap confidence intervals based on 5000 resamples were used to test the indirect effect. The indirect effect was considered statistically significant if the 95% confidence interval did not include zero. Because loneliness and depressive symptoms were both measured at follow-up, this analysis was interpreted as a half-longitudinal explanatory model rather than a strict longitudinal mediation model.

Fourth, conditional explanatory models were estimated to examine whether baseline cognitive emotion regulation strategies moderated the association between baseline physical exercise and follow-up loneliness. Two separate models were tested: one for adaptive cognitive emotion regulation strategies and one for maladaptive cognitive emotion regulation strategies. In each model, baseline physical exercise, the moderator, and their interaction term were entered as predictors of follow-up loneliness, while baseline loneliness, baseline depressive symptoms, and demographic and health-related covariates were controlled. Continuous predictors were mean-centered before creating interaction terms. Significant interactions were further examined using simple slope analyses at one standard deviation above and below the mean of the moderator. Conditional indirect effects were estimated using bootstrap procedures.

Because participants were nested within 15 communities, the potential for clustering was examined. Intraclass correlation coefficients (ICCs) were 0.032 for follow-up depressive symptoms and 0.028 for follow-up loneliness, both below the conventional 0.05 threshold typically warranting multilevel modeling. Nevertheless, to account for potential non-independence of observations within communities, robust standard errors (MLR) were used in all SEM estimations.

Model fit was evaluated using commonly recommended indices, including the chi-square statistic, comparative fit index, Tucker–Lewis index, root mean square error of approximation, and standardized root mean square residual. Values of CFI and TLI close to or above 0.90, RMSEA below 0.08, and SRMR below 0.08 were considered indicative of acceptable model fit.

### 2.6. Methodological Clarification

The present study used a two-wave observational design. Therefore, several methodological boundaries were specified in advance. First, although the study examined prospective associations, it did not establish causality. Second, although loneliness was examined as an explanatory variable linking baseline physical exercise and follow-up depressive symptoms, the mediator and outcome were measured at the same follow-up wave. Thus, the explanatory findings should be interpreted as evidence that loneliness statistically accounted for part of the prospective association, rather than as evidence of a definitive temporal mediation process. Third, cognitive emotion regulation strategies were treated as baseline moderators in the main analyses to ensure that the moderator preceded the follow-up loneliness outcome.

## 3. Results

### 3.1. Preliminary Analyses and Descriptive Statistics

The final analytic sample included 980 community-dwelling older adults who completed both the baseline and six-month follow-up surveys. Demographic characteristics of the sample are summarized in Table 1. At baseline, the mean score for physical exercise was 31.56, with a standard deviation of 16.33. The mean baseline depressive symptom score was 9.23, and the mean baseline loneliness score was 15.40. At follow-up, the mean depressive symptom score was 8.83, and the mean loneliness score was 14.95. The mean scores for baseline adaptive and maladaptive cognitive emotion regulation strategies were 28.93 and 19.03, respectively.

Based on the standard PARS-3 cut-offs, participants at baseline were classified into low (≤19; *n* = 274, 28.0%), moderate (20–42; *n* = 451, 46.0%), and high (≥43; *n* = 255, 26.0%) exercise groups. At follow-up, participants were classified into low (*n* = 281, 28.7%), moderate (*n* = 457, 46.6%), and high (*n* = 242, 24.7%) exercise groups. The distribution of exercise categories was largely stable across the two waves. Descriptive comparisons across these groups revealed a graded pattern: the low-exercise group reported the highest follow-up depressive symptoms (M = 10.62, SD = 5.11) and loneliness (M = 16.85, SD = 4.10); the moderate group reported intermediate levels (depressive symptoms M = 8.51, SD = 4.45; loneliness M = 14.62, SD = 3.65); and the high group reported the lowest levels (depressive symptoms M = 7.48, SD = 4.21; loneliness M = 13.50, SD = 3.22). Multicollinearity diagnostics indicated no concern, with all variance inflation factors below 5.0 (e.g., VIF = 1.76 for the association between follow-up loneliness and follow-up depressive symptoms).

Bivariate correlation analyses showed theoretically consistent associations among the main study variables (Table 2). Baseline physical exercise was negatively correlated with follow-up depressive symptoms and follow-up loneliness, indicating that older adults who reported higher levels of physical exercise at baseline tended to report fewer depressive symptoms and lower loneliness six months later. Follow-up loneliness was positively correlated with follow-up depressive symptoms. Baseline adaptive cognitive emotion regulation strategies were negatively correlated with follow-up loneliness and depressive symptoms, whereas baseline maladaptive cognitive emotion regulation strategies were positively correlated with both outcomes.

Specifically, baseline physical exercise was negatively associated with follow-up depressive symptoms, r = −0.452, *p* < 0.001, and follow-up loneliness, r = −0.548, *p* < 0.001. Follow-up loneliness was positively associated with follow-up depressive symptoms, r = 0.653, *p* < 0.001. Baseline adaptive strategies were negatively associated with follow-up loneliness, r = −0.549, *p* < 0.001, and follow-up depressive symptoms, r = −0.481, *p* < 0.001. In contrast, baseline maladaptive strategies were positively associated with follow-up loneliness, r = 0.565, *p* < 0.001, and follow-up depressive symptoms, r = 0.538, *p* < 0.001. These preliminary findings provided initial support for the hypothesized associations among physical exercise, loneliness, depressive symptoms, and cognitive emotion regulation strategies.

### 3.2. Prospective Association Between Physical Exercise and Depressive Symptoms

A cross-lagged analysis was conducted to examine the temporal ordering between physical exercise and depressive symptoms. The model included autoregressive paths for physical exercise and depressive symptoms, as well as cross-lagged paths from baseline physical exercise to follow-up depressive symptoms and from baseline depressive symptoms to follow-up physical exercise. Demographic, health-related, and social covariates were controlled.

The autoregressive paths were statistically significant. Baseline physical exercise positively predicted follow-up physical exercise, β = 0.466, SE = 0.031, *p* < 0.001, 95% CI [0.405, 0.527]. Baseline depressive symptoms positively predicted follow-up depressive symptoms, β = 0.551, SE = 0.026, *p* < 0.001, 95% CI [0.499, 0.603].

For the cross-lagged paths, baseline physical exercise significantly predicted lower follow-up depressive symptoms after controlling for baseline depressive symptoms and covariates, β = −0.182, SE = 0.025, *p* < 0.001, 95% CI [−0.231, −0.132]. In contrast, baseline depressive symptoms did not significantly predict follow-up physical exercise after controlling for baseline physical exercise and covariates, β = −0.016, SE = 0.033, *p* = 0.633, 95% CI [−0.080, 0.049].

**Figure 2:**
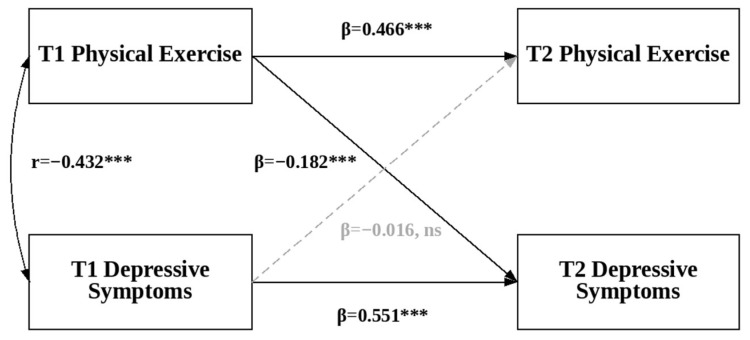
Cross-lagged associations between physical exercise and depressive symptoms. Note: *** *p* < 0.001; ns = not significant.

These findings suggest an asymmetric prospective pattern (Table 3; Figure 2). Higher baseline physical exercise was associated with lower depressive symptoms six months later, whereas baseline depressive symptoms were not significantly associated with subsequent physical exercise. Because the data were observational and limited to two waves, this asymmetric pattern should be interpreted as a residual prospective association rather than as evidence of temporal precedence or causal influence.

### 3.3. Loneliness as a Prospective Explanatory Variable

To examine whether loneliness statistically accounted for the prospective association between baseline physical exercise and follow-up depressive symptoms, a two-wave prospective explanatory model was tested. Baseline physical exercise was entered as the predictor, follow-up loneliness as the explanatory variable, and follow-up depressive symptoms as the outcome. Baseline loneliness, baseline depressive symptoms, and all covariates were controlled.

Baseline physical exercise significantly predicted lower follow-up loneliness, β = −0.267, SE = 0.024, *p* < 0.001, 95% CI [−0.314, −0.220]. Follow-up loneliness, in turn, significantly predicted higher follow-up depressive symptoms after controlling for baseline depressive symptoms, baseline loneliness, baseline physical exercise, and covariates, β = 0.324, SE = 0.033, *p* < 0.001, 95% CI [0.259, 0.388]. The direct association between baseline physical exercise and follow-up depressive symptoms remained significant after follow-up loneliness was included in the model, β = −0.075, SE = 0.026, *p* = 0.004, 95% CI [−0.126, −0.025].

**Figure 3:**
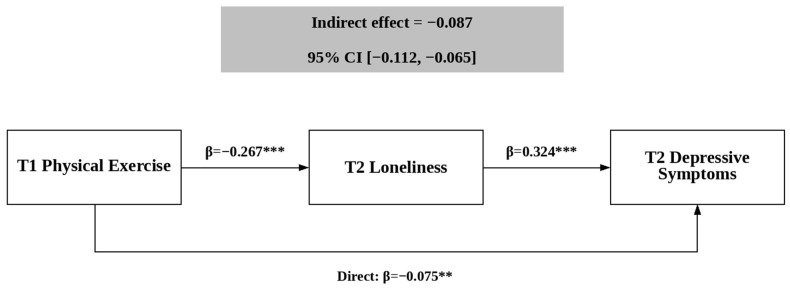
Path diagram of the two-wave prospective explanatory model linking baseline physical exercise, follow-up loneliness, and follow-up depressive symptoms. Note: ** *p* < 0.01, *** *p* < 0.001.

Bootstrap analysis indicated that the indirect association through follow-up loneliness was statistically significant (indirect effect = −0.087, 95% CI [−0.112, −0.065]), and the direct association remained significant (Table 4). In other words, baseline exercise was associated with lower follow-up loneliness, and follow-up loneliness was associated with follow-up depressive symptoms after adjustment for baseline loneliness, baseline depressive symptoms, and covariates. Because follow-up loneliness and follow-up depressive symptoms were measured concurrently, the relative size of the indirect pathway cannot be interpreted as a causal decomposition, and a proportion-mediated statistic is therefore not emphasized (Figure 3).

These results suggest that loneliness statistically accounted, in part, for the prospective association between baseline physical exercise and follow-up depressive symptoms. However, because loneliness and depressive symptoms were both measured at follow-up, this finding should be interpreted as a half-longitudinal explanatory pattern rather than as evidence of a strict temporal mediation process.

### 3.4. Moderating Role of Baseline Adaptive Cognitive Emotion Regulation Strategies

The first moderated model examined whether baseline adaptive cognitive emotion regulation strategies moderated the association between baseline physical exercise and follow-up loneliness. Baseline physical exercise, baseline adaptive strategies, their interaction term, baseline loneliness, baseline depressive symptoms, and covariates were included in the model.

Baseline physical exercise was negatively associated with follow-up loneliness, β = −0.250, SE = 0.023, *p* < 0.001, 95% CI [−0.295, −0.205] (Table 5). Baseline adaptive strategies were also negatively associated with follow-up loneliness, β = −0.183, SE = 0.024, *p* < 0.001, 95% CI [−0.231, −0.136]. Importantly, the interaction between baseline physical exercise and baseline adaptive strategies was statistically significant, β = −0.076, SE = 0.019, *p* < 0.001, 95% CI [−0.113, −0.039].

Simple slope analysis showed that baseline physical exercise was negatively associated with follow-up loneliness at both low and high levels of adaptive strategies, but the association was stronger among older adults with higher adaptive strategy use. For participants with lower adaptive strategies, the association between baseline physical exercise and follow-up loneliness was β = −0.174, SE = 0.030, *p* < 0.001, 95% CI [−0.234, −0.115]. For participants with higher adaptive strategies, the association was stronger, β = −0.326, SE = 0.030, *p* < 0.001, 95% CI [−0.384, −0.268].

Conditional indirect effect analysis showed a similar pattern. The indirect association between baseline physical exercise and follow-up depressive symptoms through follow-up loneliness was significant at both low and high levels of adaptive strategies. However, the indirect association was stronger among participants with higher adaptive strategy use. At lower levels of adaptive strategies, the conditional indirect effect was −0.056, 95% CI [−0.080, −0.035]. At higher levels of adaptive strategies, the conditional indirect effect was −0.106, 95% CI [−0.136, −0.079]. The index of the moderated explanatory effect was −0.025, 95% CI [−0.038, −0.013], indicating that adaptive cognitive emotion regulation strategies significantly strengthened the conditional indirect association involving loneliness.

These findings suggest that adaptive cognitive emotion regulation strategies amplified the association between physical exercise and lower loneliness. In other words, among older adults who used more adaptive cognitive strategies, baseline exercise was more strongly associated with a lower subsequent subjective sense of loneliness.

### 3.5. Moderating Role of Baseline Maladaptive Cognitive Emotion Regulation Strategies

The second moderated model examined whether baseline maladaptive cognitive emotion regulation strategies moderated the association between baseline physical exercise and follow-up loneliness. Baseline physical exercise, baseline maladaptive strategies, their interaction term, baseline loneliness, baseline depressive symptoms, and covariates were included in the model.

Baseline physical exercise was negatively associated with follow-up loneliness, β = −0.246, SE = 0.024, *p* < 0.001, 95% CI [−0.292, −0.199] (Table 6). Baseline maladaptive strategies were positively associated with follow-up loneliness, β = 0.158, SE = 0.026, *p* < 0.001, 95% CI [0.106, 0.210]. The interaction between baseline physical exercise and baseline maladaptive strategies was also statistically significant, β = 0.059, SE = 0.020, *p* = 0.004, 95% CI [0.019, 0.099].

Simple slope analysis indicated that baseline physical exercise was negatively associated with follow-up loneliness at both low and high levels of maladaptive strategies, but the association was weaker among older adults with higher maladaptive strategy use. For participants with lower maladaptive strategies, the association between baseline physical exercise and follow-up loneliness was β = −0.305, SE = 0.031, *p* < 0.001, 95% CI [−0.366, −0.244]. For participants with higher maladaptive strategies, the association was weaker, although still statistically significant, β = −0.186, SE = 0.031, *p* < 0.001, 95% CI [−0.248, −0.125].

**Figure 4:**
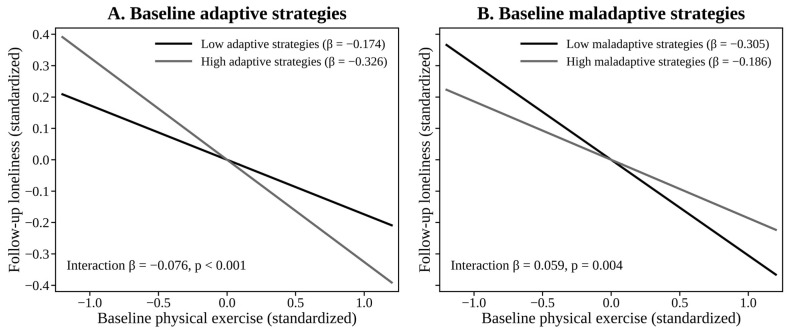
Simple slopes of the moderating effect of cognitive emotion regulation strategies.

Conditional indirect effect analysis further supported this pattern (Table 7). At lower levels of maladaptive strategies, the indirect association between baseline physical exercise and follow-up depressive symptoms through follow-up loneliness was −0.099, 95% CI [−0.130, −0.073]. At higher levels of maladaptive strategies, the conditional indirect association was reduced to −0.060, 95% CI [−0.086, −0.038]. The index of the moderated explanatory effect was 0.019, 95% CI [0.007, 0.032], indicating that maladaptive cognitive emotion regulation strategies significantly weakened the conditional indirect association involving loneliness (Figure 4).

These findings suggest that maladaptive cognitive emotion regulation strategies attenuated, but did not eliminate, the association between physical exercise and lower loneliness. Older adults who relied more heavily on maladaptive cognitive strategies appeared less likely to experience reduced loneliness in connection with physical exercise participation.

### 3.6. Sensitivity and Robustness Analyses

Several sensitivity analyses were conducted to evaluate the robustness of the findings.

First, to address the conceptual overlap between the loneliness measure and the loneliness item of the CESD-10, the main models were re-estimated after removing this item and using a 9-item depressive symptom score. The conclusions remained robust: baseline physical exercise still significantly predicted the follow-up 9-item depressive symptom score (β = −0.178, *p* < 0.001), and the indirect association through follow-up loneliness remained significant (indirect effect = −0.083, 95% CI [−0.108, −0.062]). Thus, measurement overlap did not substantially alter the findings.

Second, to test whether the association between baseline exercise and follow-up depressive symptoms was non-linear, a quadratic term for physical exercise was added to the model. The quadratic effect was not significant (β = 0.021, *p* = 0.415), supporting the use of linear specifications.

Third, to examine whether the association was disproportionately driven by participants with very low activity, two additional analyses were conducted. Re-estimating the model with ordinal exercise categories (1 = low, 2 = moderate, 3 = high) yielded a significant association with lower follow-up depressive symptoms (β = −0.165, *p* < 0.001). After excluding the low-exercise group (*n* = 274) and re-running the model among the remaining 706 participants, higher baseline exercise remained significantly associated with lower follow-up depressive symptoms (β = −0.142, *p* < 0.001). These results indicate that the associations held across the activity spectrum and were not solely attributable to the lowest-exercise subgroup.

Fourth, a progressive covariate-adjustment strategy was used to assess the stability of the main prospective association across model specifications. Baseline exercise predicted lower follow-up depressive symptoms in the unadjusted model (Model 1: β = −0.215, *p* < 0.001) and remained stable after sequentially adjusting for demographic variables (Model 2: β = −0.198, *p* < 0.001), health-related covariates (Model 3: β = −0.189, *p* < 0.001), and social variables, including social activity frequency and interpersonal support (Model 4: β = −0.182, *p* < 0.001). The stability of the coefficient indicates that the association was neither spuriously generated nor eliminated by adjustment for social variables, which may lie partly on the hypothesized pathway.

Fifth, to confirm the robustness of the moderation effects, an integrated model simultaneously including both moderators and their interaction terms with baseline physical exercise was estimated. Both interactions remained significant (exercise × adaptive strategies: β = −0.065, *p* = 0.002; exercise × maladaptive strategies: β = 0.048, *p* = 0.015), and the interaction terms accounted for a small but significant increment in explained variance (adaptive: ΔR^2^ = 0.015, *p* < 0.001; maladaptive: ΔR^2^ = 0.008, *p* = 0.004). This indicates that adaptive and maladaptive strategies operated as independent boundary conditions for the exercise–loneliness association.

Across all model specifications, the overall pattern of results was consistent. The fit of the cross-lagged model (χ^2^/df = 2.15, CFI = 0.978, TLI = 0.965, RMSEA = 0.042, SRMR = 0.038) and the explanatory model (χ^2^/df = 2.31, CFI = 0.965, TLI = 0.952, RMSEA = 0.048, SRMR = 0.041) was good.

## 4. Discussion

### 4.1. Principal Findings

This two-wave prospective study examined the association between self-reported physical exercise and depressive symptoms among community-dwelling older adults, with particular attention to the explanatory role of loneliness and the moderating role of baseline cognitive emotion regulation strategies. Three main findings emerged.

First, baseline physical exercise was significantly associated with lower depressive symptoms six months later after controlling for baseline depressive symptoms and relevant covariates. In contrast, baseline depressive symptoms were not significantly associated with subsequent physical exercise after baseline exercise was controlled. This asymmetric pattern suggests that baseline exercise was associated with residual differences in depressive symptoms six months later. Given the observational, two-wave nature of the data, this finding should be interpreted as a prospective association rather than as evidence of temporal precedence or a causal effect.

Second, follow-up loneliness partially accounted for the association between baseline physical exercise and follow-up depressive symptoms. Older adults who reported higher levels of physical exercise at baseline tended to report lower loneliness at follow-up, and lower loneliness was in turn associated with fewer depressive symptoms. This finding suggests that loneliness may be an important psychosocial correlate associated with the link between physical exercise participation and depressive symptoms. Nevertheless, because loneliness and depressive symptoms were both measured at follow-up, this result should be understood as a half-longitudinal explanatory pattern rather than a strict temporal mediation process.

Third, baseline cognitive emotion regulation strategies moderated the association between physical exercise and loneliness. Adaptive strategies strengthened the negative association between baseline physical exercise and follow-up loneliness, whereas maladaptive strategies weakened this association. These findings indicate that physical exercise may not operate in isolation. Rather, its psychological relevance may depend partly on how older adults cognitively process exercise-related social and emotional experiences.

Together, these findings support a more nuanced understanding of physical exercise in later life. Exercise is not only a physical behavior but may also occur within psychosocial contexts in which behavioral activation, social contact, self-regulation, and cognitive appraisal jointly shape mental health outcomes. Because these processes were not directly measured, they should be interpreted as plausible mechanisms for future research rather than as processes demonstrated by the present data.

### 4.2. Physical Exercise and Subsequent Depressive Symptoms

The finding that baseline physical exercise was associated with lower depressive symptoms six months later is consistent with previous prospective and intervention-based evidence suggesting that physical activity is beneficial for depressive symptoms and depression risk. For example, Schuch et al. (2018) reported in a meta-analysis of prospective cohort studies that higher physical activity was associated with reduced risk of incident depression across age groups and geographical regions. Exercise has also been considered a potentially valuable strategy for older adults because it is comparatively accessible, low-cost, and compatible with community health promotion (Solis-Urra et al., 2024).

From a sports psychology perspective, the association between physical exercise and later depressive symptoms can be interpreted through several complementary but partly unmeasured mechanisms. First, physical exercise may provide a form of behavioral activation. Behavioral activation theory emphasizes that engagement in structured, meaningful, and reinforcing activities can interrupt withdrawal and inactivity, which are central behavioral features of depression (Jacobson et al., 2001). For older adults, retirement, bereavement, chronic illness, and reduced family roles may weaken daily structure and reduce opportunities for positive reinforcement. Physical exercise may help restore routine, goal-directed activity, and a sense of behavioral agency.

Second, physical exercise may support perceived competence and self-efficacy. Older adults who participate in exercise may experience improvements in physical function, energy, balance, or endurance. Even modest perceived improvements can provide mastery experiences, which may buffer feelings of helplessness and reinforce more active engagement with daily life (Bandura, 1977; Teixeira et al., 2012). This is especially important in later life, when declining physical capacity can threaten autonomy and self-worth.

Third, exercise may also carry social meaning when it takes place in group or community settings. Activities such as square dancing, group walking, tai chi, or community fitness sessions can provide repeated contact with familiar others (Wang et al., 2024), which may help older adults maintain a sense of social rhythm and belonging. However, because the present measure assessed overall exercise volume and did not record the social setting of exercise, this social interpretation should be regarded as a plausible but untested mechanism rather than a process directly demonstrated by the data.

It is also noteworthy that the reverse path from baseline depressive symptoms to follow-up physical exercise was not significant. This result does not mean that depressive symptoms never influence physical activity. Depression is often associated with fatigue, low motivation, and withdrawal, all of which may reduce physical activity in other contexts. However, in the present sample, the predictive contribution of baseline depressive symptoms to later exercise was not evident after baseline exercise and covariates were controlled. One possible explanation is that some forms of regular exercise among older adults may be partly sustained by habit, peer expectations, and social routines. Because the present study did not measure these processes directly, this interpretation should be treated cautiously.

### 4.3. Loneliness as a Psychosocial Explanatory Mechanism

The present study found that loneliness partially accounted for the prospective association between physical exercise and depressive symptoms. This finding is theoretically meaningful because loneliness is one of the most important social–emotional risk factors in later life. Loneliness is not equivalent to objective social isolation; rather, it reflects the subjective perception that one’s social relationships are insufficient in quantity or quality. This distinction is important because older adults may feel lonely even when they are not physically alone, and conversely may not feel lonely when they have relatively small but emotionally meaningful networks (Lee & Schafer, 2025).

Previous research has consistently linked loneliness with depressive symptoms. Cacioppo et al. (2006) showed that loneliness is a specific risk factor for depressive symptoms in middle-aged and older adults, while Erzen and Çikrikci (2018) reported a significant association between loneliness and depression in their meta-analysis. Longitudinal reviews have also suggested that loneliness and depressive symptoms are closely connected over time, although their relationship can be reciprocal rather than strictly unidirectional.

The current findings extend this literature by situating loneliness within the context of physical exercise participation. Physical exercise may be associated with lower loneliness through several processes, although these processes were not directly measured in the present study. At the behavioral level, exercise increases exposure to shared spaces and repeated encounters. Repeated contact is important because social connection often develops gradually through familiarity, informal conversation, and mutual recognition. At the emotional level, exercising with others may generate shared positive affect, companionship, and a sense of group identity (Stevens et al., 2017). At the role level, regular participation may allow older adults to see themselves not merely as care recipients or retired individuals, but as active members of a community.

This mechanism is especially relevant in later life. According to socioemotional selectivity theory, older adults become increasingly selective in their social goals and tend to prioritize emotionally meaningful relationships as perceived time horizons become limited (Carstensen et al., 1999). This motivational shift can support emotional well-being when meaningful ties are available, but it may also increase vulnerability when close relationships are lost or social networks shrink. Exercise in group or community settings may offer a relatively low-pressure environment for maintaining meaningful, repeated, and emotionally positive contact. In this sense, the exercise–loneliness association may reflect not only a behavioral process but also a social–motivational process, although the present data do not directly test the social setting of exercise.

At the same time, the present findings should be interpreted with appropriate caution. Although baseline loneliness was controlled, loneliness and depressive symptoms were measured at the same follow-up wave. Therefore, the observed association between follow-up loneliness and follow-up depressive symptoms cannot establish that loneliness temporally preceded depressive symptoms. The reverse direction is plausible: older adults with more depressive symptoms may perceive social relationships more negatively, withdraw from interaction, or report stronger loneliness. Thus, loneliness should be described as a psychosocial explanatory variable that statistically accounted for part of the association between baseline exercise and follow-up depressive symptoms, not as a confirmed causal mediator. This interpretation is consistent with methodological recommendations that rigorous longitudinal mediation ideally requires at least three waves of data with temporally separated predictor, mediator, and outcome variables (Cole & Maxwell, 2003).

### 4.4. Cognitive Emotion Regulation as a Boundary Condition

One of the most important contributions of this study is the finding that baseline cognitive emotion regulation strategies moderated the association between physical exercise and loneliness. This result may help account for why the same behavioral exposure—participation in physical exercise—may not be associated with the same psychological experience for all older adults.

Adaptive cognitive emotion regulation strategies strengthened the negative association between physical exercise and follow-up loneliness. Older adults with higher levels of adaptive strategies may be better able to extract social and emotional benefits from exercise-related experiences. For example, positive reappraisal may help them interpret exercise-related discomfort or interpersonal awkwardness as normal and manageable. Positive refocusing may direct attention toward enjoyable aspects of the activity. Refocus on planning may help them maintain participation despite temporary barriers. Putting into perspective may prevent minor social difficulties from being overinterpreted. In this way, adaptive strategies may help transform objective participation into subjective connection.

By contrast, maladaptive cognitive emotion regulation strategies weakened the association between physical exercise and lower loneliness. Older adults who frequently use rumination, self-blame, other-blame, or catastrophizing may interpret ambiguous social cues more negatively. A brief lack of response from another participant may be perceived as rejection; a minor mistake during exercise may be interpreted as embarrassment; an ordinary interpersonal disagreement may be magnified into a reason for withdrawal. These cognitive patterns may reduce the likelihood that exercise-related experiences are perceived as socially rewarding. As a result, even when such individuals participate in exercise, the subjective reduction in loneliness may be weaker.

This finding is consistent with the broader emotion regulation literature. The CERQ-short preserves nine cognitive emotion regulation dimensions, including adaptive and maladaptive strategies such as positive reappraisal, planning, rumination, self-blame, and catastrophizing (Garnefski & Kraaij, 2006). Meta-analytic evidence also indicates that maladaptive strategies, particularly rumination and avoidance-related processes, are more strongly associated with psychopathological symptoms, whereas adaptive strategies such as reappraisal and acceptance tend to show weaker but generally protective associations (Aldao et al., 2010). The present study extends this literature to the context of older adults’ physical exercise, suggesting that cognitive emotion regulation may shape the emotional meaning of exercise-related social experiences.

Importantly, the moderating effects were observed using baseline cognitive emotion regulation strategies. This strengthens the temporal logic of the model compared with using follow-up cognitive strategies as moderators. Conceptually, cognitive emotion regulation strategies can be understood as relatively stable tendencies in how individuals process emotional information. When measured at baseline, they are better positioned as boundary conditions that precede the follow-up loneliness outcome. This modeling choice also reduces the ambiguity that would arise if the moderator and mediator were assessed at the same time point.

These findings have theoretical significance. They suggest that physical exercise should not be understood simply as a “dose” of activity. Instead, its psychological implications may depend on both environmental opportunities and individual cognitive processing. Exercise may provide social opportunities in some settings, but cognitive emotion regulation is associated with whether those opportunities are encoded as connection, threat, embarrassment, or indifference. Thus, the present study integrates behavioral, social–emotional, and cognitive levels of analysis in understanding depressive symptoms among older adults.

### 4.5. Theoretical Implications

This study contributes to the literature in three ways.

First, it provides prospective evidence for the association between physical exercise and depressive symptoms among older adults. By controlling for baseline depressive symptoms and testing the reverse path, the study offers a more informative examination of prospective ordering than cross-sectional research, although two-wave designs remain limited for establishing developmental or within-person processes. The asymmetric prospective pattern supports the view that physical exercise is a meaningful behavioral correlate of later depressive symptoms.

Second, the study highlights loneliness as a central psychosocial process. Many studies have examined the direct association between physical exercise and depressive symptoms, but fewer have examined how social–emotional experiences may be associated with this link. The present findings suggest that the mental health relevance of physical exercise may partly depend on its association with lower subjective social disconnection. This shifts attention from exercise as a purely physiological intervention to exercise as a socially embedded activity.

Third, the study identifies cognitive emotion regulation as a boundary condition. This is important because exercise interventions are often designed under the assumption that increasing participation will automatically improve psychological well-being. The present findings suggest that this assumption is incomplete. Older adults may differ in their ability to psychologically benefit from the same exercise context. Cognitive strategies may be associated with whether exercise-related social exposure is experienced as emotionally nourishing or as psychologically neutral. This insight advances a more person-centered model of exercise and mental health in later life.

### 4.6. Practical Implications

The findings suggest several hypotheses for the future development and testing of community-based mental health promotion among older adults. Because the relevant program features were not manipulated or measured in this observational study, the following points should be regarded as suggestions for future intervention research rather than recommendations directly demonstrated by the present data.

First, future intervention studies could examine whether exercise programs that increase physical activity volume and deliberately enhance social connection yield stronger mental-health benefits than activity-only formats. Program organizers could test formats that promote repeated interaction, cooperation, mutual recognition, and group belonging, such as small-group walking, tai chi groups, dance groups, peer-led warm-up routines, and post-exercise social activities.

Second, future community-health programs could evaluate loneliness as a potential intervention target. Screening for loneliness may help identify older adults who are at elevated psychological risk even if they are already participating in physical activity. For such individuals, merely increasing exercise frequency may not be sufficient; structured opportunities for interpersonal engagement, peer support, and inclusion may warrant formal testing.

Third, the moderating role of cognitive emotion regulation suggests that integrated interventions may be worth testing for some older adults. Those who rely heavily on maladaptive cognitive strategies may require additional psychological support to benefit fully from exercise-related social experiences. Brief cognitive–behavioral components, such as reducing rumination, challenging catastrophic interpretations, encouraging positive reappraisal, and strengthening planning-based coping, might be incorporated into community exercise programs and evaluated in future trials. These components need not be highly clinical or intensive. Simple group-based reflection, supportive feedback, and guided reframing may help older adults process exercise-related experiences more adaptively.

Fourth, the findings raise the possibility of a precision-oriented approach to exercise promotion. Older adults with high adaptive and low maladaptive cognitive emotion regulation may respond well to standard exercise programs, whereas those with high maladaptive cognitive strategy use may need more structured social support and cognitive guidance. Such stratification should be evaluated empirically before being implemented as a practice recommendation.

### 4.7. Limitations and Future Directions

Several limitations should be acknowledged.

First, the study used a two-wave observational design. Although baseline physical exercise predicted follow-up depressive symptoms and the reverse path was not significant, causal conclusions cannot be drawn. Unmeasured variables, such as personality, life events, sleep quality, pain, medication use, or neighborhood resources, may partly explain the observed associations. Future studies should use randomized controlled trials or natural experiments to test whether increasing physical exercise, especially exercise in explicitly social settings, leads to reductions in loneliness and depressive symptoms.

Second, the explanatory analysis was half-longitudinal rather than fully longitudinal. Loneliness and depressive symptoms were both assessed at follow-up. Although baseline loneliness and baseline depressive symptoms were controlled, the temporal ordering between follow-up loneliness and follow-up depressive symptoms could not be established. Future research should use at least three waves of data, for example physical exercise at T1, loneliness at T2, and depressive symptoms at T3, to more rigorously evaluate the temporal sequence implied by mediation.

Third, the use of a traditional cross-lagged panel approach has methodological limitations. Traditional CLPMs cannot fully separate within-person change processes from stable between-person differences. Hamaker et al. argued that random-intercept cross-lagged panel models can better distinguish stable trait-like differences from within-person fluctuations (Hamaker et al., 2015). Because the present study had only two waves, RI-CLPM could not be appropriately estimated. Future studies with three or more waves should consider RI-CLPM or latent growth models to clarify whether changes in exercise are associated with changes in loneliness and depressive symptoms within individuals over time.

Fourth, all key variables were assessed by self-report questionnaires. Although the measures showed acceptable reliability, self-report data may be influenced by recall bias, common method variance, social desirability, and response-mode differences. These issues are particularly relevant when physical exercise, loneliness, cognitive emotion regulation, and depressive symptoms are all reported by the same participants. Future studies should combine self-report measures with objective indicators, such as accelerometry-based physical activity, clinician-rated depressive symptoms, ecological momentary assessment of loneliness, or observational data on social interaction during exercise sessions.

Fifth, physical exercise was assessed as an overall exercise score and did not distinguish different types of activity. This distinction may be theoretically important. Group-based exercise, solitary walking, household activity, and structured fitness training may have different implications for loneliness and depressive symptoms. Future studies should examine whether socially interactive forms of exercise are more strongly associated with loneliness reduction than solitary forms.

Sixth, the sample was drawn from selected urban communities in Zhengzhou through community-based recruitment. Although the study provides useful evidence for community-dwelling older adults in an urban Chinese context, the findings should not be generalized uncritically to rural older adults, institutionalized older adults, older adults from other regions, or older adults from different cultural settings. Future research should test the model in more diverse samples and examine whether community structure, family norms, and access to public exercise spaces influence the observed associations.

Finally, the present study focused on loneliness and cognitive emotion regulation, but other mechanisms may also be involved. Future research should examine parallel or sequential processes including sleep quality, perceived stress, self-efficacy, physical function, inflammatory markers, and social support. A more comprehensive model may clarify how biological, behavioral, social, and cognitive processes jointly link physical exercise with mental health in later life.

## 5. Conclusions

In conclusion, self-reported physical exercise was prospectively associated with fewer depressive symptoms among older adults six months later. This association was statistically accounted for, in part, by lower levels of loneliness at follow-up. Furthermore, individual differences in cognitive emotion regulation were associated with the strength of the exercise–loneliness association: adaptive strategies amplified the inverse association between exercise and loneliness, whereas maladaptive strategies attenuated it. Because the explanatory variable and outcome were measured at the same follow-up wave, these findings document a prospective explanatory pathway rather than a strict temporal mediation, and future research employing three or more waves is needed to isolate the chronological sequence of these psychosocial processes.

## Figures and Tables

**Table 1:** Demographic characteristics of participants (*N* = 980).

Variable	Category	*n*	%
Gender	Male	424	43.3
	Female	556	56.7
Age group (years)	60–69	333	34.0
	70–79	506	51.6
	≥80	141	14.4
Education level	Primary school and below	424	43.3
	Junior high	325	33.2
	High school/Vocational school	179	18.3
	Junior college and above	52	5.3
Living arrangement	Living alone	159	16.2
	Living with spouse	515	52.6
	Living with children	184	18.8
	Living with spouse and children	108	11.0
	Other	14	1.4
Marital status	Single/Separated/Widowed/Divorced	260	26.5
	Married	720	73.5
Smoking	No	910	92.9
	Yes	70	7.1
Alcohol consumption	No	898	91.6
	Yes	82	8.4
Number of chronic diseases	0	389	39.7
	1–2	535	54.6
	≥3	56	5.7
Self-rated health status	Very good/Good	452	46.1
	Fair	416	42.4
	Poor/Very poor	112	11.4
Social activity frequency	None/Very little	222	22.7
	Little	187	19.1
	Moderate	253	25.8
	Much/Very much	318	32.4
Interpersonal support	Never/Rarely	100	10.2
	Sometimes	227	23.2
	Most of the time	412	42.0
	Always	241	24.6

**Table 2:** Descriptive statistics and correlations among study variables.

Variable	M	SD	1	2	3	4	5	6	7	8	9	10
1. T1 Physical exercise	31.56	16.33	—									
2. T1 Depressive symptoms	9.23	4.96	−0.432 ***	—								
3. T1 Loneliness	15.40	4.19	−0.447 ***	0.670 ***	—							
4. T1 Adaptive strategies	28.93	7.03	0.346 ***	−0.504 ***	−0.518 ***	—						
5. T1 Maladaptive strategies	19.03	5.71	−0.390 ***	0.573 ***	0.553 ***	−0.386 ***	—					
6. T2 Physical exercise	31.06	16.05	0.502 ***	−0.241 ***	−0.281 ***	0.175 ***	−0.200 ***	—				
7. T2 Depressive symptoms	8.83	4.78	−0.452 ***	0.698 ***	0.572 ***	−0.481 ***	0.538 ***	−0.230 ***	—			
8. T2 Loneliness	14.95	3.92	−0.548 ***	0.601 ***	0.698 ***	−0.549 ***	0.565 ***	−0.287 ***	0.653 ***	—		
9. T2 Adaptive strategies	29.47	6.79	0.432 ***	−0.531 ***	−0.525 ***	0.931 ***	−0.412 ***	0.223 ***	−0.493 ***	−0.567 ***	—	
10. T2 Maladaptive strategies	18.59	5.57	−0.409 ***	0.597 ***	0.556 ***	−0.399 ***	0.937 ***	−0.203 ***	0.532 ***	0.554 ***	−0.427 ***	—

Note: *** *p* < 0.001.

**Table 3:** Cross-lagged/prospective associations between physical exercise and depressive symptoms.

Outcome	Predictor	β	SE	t	*p*	95% CI	R^2^
T2 depressive symptoms	T1 Depressive symptoms	0.551	0.026	20.793	<0.001	[0.499, 0.603]	0.541
T2 depressive symptoms	T1 Physical exercise	−0.182	0.025	−7.251	<0.001	[−0.231, −0.132]	0.541
T2 physical exercise	T1 Physical exercise	0.466	0.031	15.008	<0.001	[0.405, 0.527]	0.293
T2 physical exercise	T1 Depressive symptoms	−0.016	0.033	−0.478	0.633	[−0.080, 0.049]	0.293

**Table 4:** Prospective explanatory role of loneliness.

Path/Effect	β/Effect	SE	*p*	95% CI
T1 physical exercise → T2 loneliness	−0.267	0.024	<0.001	[−0.314, −0.220]
T2 loneliness → T2 depressive symptoms	0.324	0.033	<0.001	[0.259, 0.388]
Direct effect: T1 physical exercise → T2 depressive symptoms	−0.075	0.026	0.004	[−0.126, −0.025]
Indirect effect	−0.087	0.012	CI excludes 0	[−0.112, −0.065]
Total effect	−0.162	0.025	CI excludes 0	[−0.210, −0.113]

Note: Standardized coefficients are reported. The indirect, direct, and total effects were estimated using bootstrap procedures with 5000 resamples. Models controlled for baseline depressive symptoms; baseline loneliness; and demographic, health-related, and social covariates. Because T2 loneliness and T2 depressive symptoms were measured at the same wave, this table should be viewed as a two-wave prospective or half-longitudinal explanatory model, not as a strict longitudinal mediation model. The proportion of the association attributable to the indirect pathway is not reported, as such a decomposition cannot be justified when the explanatory variable and outcome are measured concurrently.

**Table 5:** Regression results for the moderating effect of baseline adaptive cognitive emotion regulation strategies on the physical exercise–loneliness association.

Predictor	β	SE	t	*p*	95% CI	Model R^2^/F
T1 physical exercise	−0.250	0.023	−10.820	<0.001	[−0.295, −0.205]	R^2^ = 0.630; F = 59.91
Adaptive strategies	−0.183	0.024	−7.570	<0.001	[−0.231, −0.136]	R^2^ = 0.630; F = 59.91
T1 physical exercise × Adaptive strategies	−0.076	0.019	−3.993	<0.001	[−0.113, −0.039]	R^2^ = 0.630; F = 59.91
Low adaptive strategies (M − 1 SD)	−0.174	0.030	−5.753	<0.001	[−0.234, −0.115]	
High adaptive strategies (M + 1 SD)	−0.326	0.030	−11.014	<0.001	[−0.384, −0.268]	

Note: Outcome = T2 loneliness. Standardized coefficients are reported. Low and high adaptive strategies refer to one standard deviation below and above the mean. The model controlled for baseline loneliness; baseline depressive symptoms; and demographic, health-related, and social covariates.

**Table 6:** Regression results for the moderating effect of baseline maladaptive cognitive emotion regulation strategies on the physical exercise–loneliness association.

Predictor	β	SE	t	*p*	95% CI	Model R^2^/F
T1 physical exercise	−0.246	0.024	−10.388	<0.001	[−0.292, −0.199]	R^2^ = 0.618; F = 56.95
Maladaptive strategies	0.158	0.026	5.992	<0.001	[0.106, 0.210]	R^2^ = 0.618; F = 56.95
T1 physical exercise × Maladaptive strategies	0.059	0.020	2.919	0.004	[0.019, 0.099]	R^2^ = 0.618; F = 56.95
Low maladaptive strategies (M − 1 SD)	−0.305	0.031	−9.848	<0.001	[−0.366, −0.244]	
High maladaptive strategies (M + 1 SD)	−0.186	0.031	−5.931	<0.001	[−0.248, −0.125]	

Note: Outcome = T2 loneliness. Standardized coefficients are reported. Low and high maladaptive strategies refer to one standard deviation below and above the mean. The model controlled for baseline loneliness; baseline depressive symptoms; and demographic, health-related, and social covariates.

**Table 7:** Conditional indirect effects at different levels of cognitive emotion regulation strategies.

Moderator	Level	Conditional Indirect Effect	SE	95% CI
Adaptive strategies	Low (M − 1 SD)	−0.056	0.012	[−0.080, −0.035]
Adaptive strategies	High (M + 1 SD)	−0.106	0.014	[−0.136, −0.079]
Adaptive strategies	Index of moderated explanatory effect	−0.025	0.006	[−0.038, −0.013]
Maladaptive strategies	Low (M − 1 SD)	−0.099	0.014	[−0.130, −0.073]
Maladaptive strategies	High (M + 1 SD)	−0.060	0.012	[−0.086, −0.038]
Maladaptive strategies	Index of moderated explanatory effect	0.019	0.006	[0.007, 0.032]

Note: Bootstrap confidence intervals were estimated using 5000 resamples. Confidence intervals that do not include zero indicate statistically significant conditional indirect effects. The indirect pathway refers to T1 physical exercise → T2 loneliness → T2 depressive symptoms. Because loneliness and depressive symptoms were measured at the same follow-up wave, these effects should be interpreted as conditional half-longitudinal explanatory effects rather than strict longitudinal mediation effects.

## Data Availability

The datasets presented in this article are not readily available because of privacy and ethical restrictions regarding the participants’ sensitive information. Requests to access the datasets should be directed to the corresponding author.

## Supplementary Materials

The following supporting information can be downloaded at: https://www.mdpi.com/article/10.3390/bs16071108/s1. Table S1: STROBE checklist for cohort studies.
