# Supplementary material for: Longitudinal Association Between Physical Exercise and Depressive Symptoms in Older Adults: The Prospective Explanatory Role of Loneliness and the Moderating Role of Cognitive Emotion Regulation
Source: Behav Sci (Basel). 2026 Jul 3;16(7):1108. doi: 10.3390/bs16071108 (PMC13405692; doi:10.3390/bs16071108)
Supplement: Supplementary file 1 [file behavsci-16-01108-s001.zip › behavsci-4401850-supplementary.pdf]

Table S1. STROBE checklist for cohort studies

STROBE Statement—Checklist of items that should be included in reports of cohort studies

Manuscript: behavsci-4401850 — Longitudinal Association Between Physical Exercise and Depressive Symptoms in Older Adults

**Topic**	**Item No**	**Recommendation**	**Reported in (page/section)**
**Introduction**			
Title and abstract	1	(a) Indicate the study's design with a commonly used term in the title or the abstract	**Abstract ("two-wave prospective survey")**
Title and abstract	1	(b) Provide in the abstract an informative and balanced summary of what was done and what was found	**Abstract**
**Methods**			
Background/rationale	2	Explain the scientific background and rationale for the investigation being reported	**Introduction (Section 1)**
Objectives	3	State specific objectives, including any prespecified hypotheses	**Introduction (Section 1), final paragraph (three research questions)**
Study design	4	Present key elements of study design early in the paper	**Section 2.1, Study Design**
Setting	5	Describe the setting, locations, and relevant dates, including periods of recruitment, exposure, follow-up, and data collection	**Section 2.1, Setting (Zhengzhou; baseline Sept 2024, follow-up Mar 2025)**
Participants	6	(a) Give the eligibility criteria, and the sources and methods of selection of participants. Describe methods of follow-up	**Section 2.1, Participants (Eligibility Criteria) and Participant Flow**
Participants	6	(b) For matched studies, give matching criteria and number of exposed and unexposed	**Not applicable (not a matched cohort study)**
Variables	7	Clearly define all outcomes, exposures, predictors, potential confounders, and effect modifiers. Give diagnostic criteria, if applicable	**Section 2.3 (Measures); covariates in Section 2.1 and 2.3.1**
Data sources/ measurement	8*	For each variable of interest, give sources of data and details of methods of assessment (measurement). Describe comparability of assessment methods if there is more than one group	**Section 2.3 (Measures), Section 2.4 (Data Collection Procedure)**

Bias	9	Describe any efforts to address potential sources of bias	**Section 2.1, Bias**
Study size	10	Explain how the study size was arrived at	**Section 2.2 (Sample Size Consideration / Study Size)**
Quantitative variables	11	Explain how quantitative variables were handled in the analyses. If applicable, describe which groupings were chosen and why	**Section 2.5 (continuous scores); Section 3.1 (PARS-3 low/moderate/high categories)**
Statistical methods	12	(a) Describe all statistical methods, including those used to control for confounding	**Section 2.5 (Statistical Analysis / Statistical Methods)**
Statistical methods	12	(b) Describe any methods used to examine subgroups and interactions	**Section 2.5; Sections 3.4–3.6 (moderation, integrated model)**
Statistical methods	12	(c) Explain how missing data were addressed	**Section 2.1 (FIML); Section 2.5**
Statistical methods	12	(d) If applicable, explain how loss to follow-up was addressed	**Section 2.1 (attrition analysis with effect sizes; FIML)**
Statistical methods	12	(e) Describe any sensitivity analyses	**Section 2.5; Section 3.6 (Sensitivity and Robustness Analyses)**
**Results**			
Participants	13*	(a) Report numbers of individuals at each stage of study	**Section 2.1, Participant Flow (1,350 invited → 1,200 baseline → 980 analysed)**
Participants	13*	(b) Give reasons for non-participation at each stage	**Section 2.1, Participant Flow**
Participants	13*	(c) Consider use of a flow diagram	**Section 2.1 (participant flow reported in text)**
Descriptive data	14*	(a) Give characteristics of study participants and information on exposures and potential confounders	**Section 3.1; Table 1**
Descriptive data	14*	(b) Indicate number of participants with missing data for each variable of interest	**Section 2.1 (FIML; complete data on main variables, N = 980)**
Descriptive data	14*	(c) Summarise follow-up time	**Section 2.1 (six-month interval; Sept 2024–Mar 2025)**
Outcome data	15*	Report numbers of outcome events or summary measures over time	**Section 3.1 (means/SD of depressive symptoms at both waves); Table 1**
Main results	16	(a) Give unadjusted estimates and confounder-adjusted estimates and their precision (eg 95% CI). Make clear which confounders were adjusted for and why	**Sections 3.2–3.5; Section 3.6 (progressive covariate-adjusted models, Models 1–4, with 95% CIs)**
Main results	16	(b) Report category boundaries when continuous variables were categorized	**Section 3.1 (PARS-3 cut-offs: low ≤19, moderate 20–42, high ≥43)**

Main results	16	(c) If relevant, consider translating estimates of relative risk into absolute risk	**Not applicable**
Other analyses	17	Report other analyses done—eg subgroups, interactions, sensitivity analyses	**Section 3.6 (Sensitivity and Robustness Analyses)**
**Discussion**			
Key results	18	Summarise key results with reference to study objectives	**Section 4.1 (Principal Findings)**
Limitations	19	Discuss limitations, taking into account sources of potential bias or imprecision	**Section 4.7 (Limitations and Future Directions)**
Interpretation	20	Give a cautious overall interpretation of results	**Section 4 (Discussion); Section 5 (Conclusion)**
Generalisability	21	Discuss the generalisability (external validity) of the study results	**Section 4.7 (Limitations and Future Directions)**
**Other information**			
Funding	22	Give the source of funding and the role of the funders	**Funding statement (end of manuscript)**

*Note. *Give such information separately for exposed and unexposed groups (not applicable to the present single-cohort design). Section references correspond to the revised manuscript; exact page numbers can be added after final typesetting.*
